# Supplementary material for: South-American plate advance and forced Andean trench retreat as drivers for transient flat subduction episodes
Source: Nat Commun. 2017 May 16;8:15249. doi: 10.1038/ncomms15249 (PMC5440808; doi:10.1038/ncomms15249)
Supplement: Supplementary Information — Supplementary Table and Supplementary References [file ncomms15249-s1.pdf]

**Supplementary Table 1. Shortening estimates used in the reconstruction of the Andes. See Figure 1 for section locations.**

| Structure/Region | Transect | Type of data | Sense | Amount | Plate ID | Age | Reference |
|---|---|---|---|---|---|---|---|
| **Subandes** | 1 | Shortening | e-w | 11 km | 2551 | 15-0 Ma | 1 |
| | 2 | Shortening | e-w | 20 km | 2551 | 15-0 Ma | 2, 3 |
| | 3 | Shortening | ne-sw | 84 km | 2553 | 24-0 Ma | 4, 5 |
| | 4 | Shortening | ne-sw | 95 km | 2553 | 24-0 Ma | 5 |
| | 5 | Shortening | ne-sw | 24,7 km | 2552 | 24-0 Ma | 5, 6 |
| | 6 | Shortening | ne-sw | 40 km | 2546 | 24-0 Ma | 3, 5, 7 |
| | 7 | Shortening | ne-sw | 42,3 km | 2546 | 24-0 Ma | 5, 6 |
| | 8 | Shortening | ne-sw | 56 km | 2593 | 15-0 Ma | 4, 8 |
| | 9 | Shortening | ne-sw | 17 km | 2544 | 15-0 Ma | 9 |
| | 10 | Shortening | ne-sw | 40 km | 2544 | 15-0 Ma | 1, 9 |
| | 11 | Shortening | ne-sw | 20 km | 2544 | 15-0 Ma | 3, 9, 10 |
| | 12 | Shortening | ne-sw | 105 km | 2539 | 15-0 Ma | 11, 12, 13 |
| | 13 | Shortening | ne-sw | 135 km | 2539 | 15-0 Ma | 13, 14 |
| | 14 | Shortening | ne-sw | 66 km | 2536 | 15-0 Ma | 13 |
| | 15 | Shortening | ne-sw | 74 km | 2536 | 15-0 Ma | 13, 15 |
| | 16 | Shortening | ne-sw | 72 km | 2533 | 10-0 Ma | 16, 17 |
| | 18 | Shortening | ene-wsw | 86 km | 2530 | 15-0 Ma | 18 |
| | 19 | Shortening | e-w | 67 km | 2523 | 15-0 Ma | 16, 19 |
| | 21 | Shortening | e-w | 60-78 km | 2519 | 15-0 Ma | 20, 21 |
| | 22 | Shortening | e-w | 60 km | 2519 | 9-0 Ma | 22 |
| | | Rotation | ccw | 13 degrees | 2530, 36, 39 | 15-0 Ma | 23 |
| | | Rotation | ccw | 13 degrees | 2533 | 10-0 Ma | 23 |
| | | Rotation | cw | 13 degrees | 2519, 23, 28 | 15-0 Ma | 23 |

| | | | | | | | |
|---|---|---|---|---|---|---|---|
| **Eastern Cordillera** | 4 | Shortening | ne-sw | 47 km | 2549 | 30-23 Ma | 5 |
| | 5 | Shortening | ne-sw | 26 km | 2547 | 30-20 Ma | 5, 6 |
| | 7 | Shortening | ne-sw | 46 km | 2554 | 30-20 Ma | 5, 6 |
| | 9 | Shortening | ne-sw | 91 km | 2596 | 45-20 Ma | 9 |
| | 10 | Shortening | ne-sw | 81 km | 2596 | 45-20 Ma | 1, 9 |
| | 14 | Shortening | ne-sw | 123 km | 2538 | 50-25 Ma | 13, 24 |
| | 16 | Shortening | ne-sw | 142 km | 2535 | 50-25 Ma | 16, 17 |
| | 17 | Shortening | ene-wsw | 136 km | 2532 | 50-25 Ma | 18 |
| | 19 | Shortening | e-w | 122 km | 2525 | 40-20 Ma | 16, 19 |
| | 21 | Shortening | e-w | 95 km | 2522 | 40-20 Ma | 25, 26, 27 |
| | 27 | Shortening | e-w | 70 km | 2594 | 40-20 Ma | 28, 29, 30 |
| **Interandes** | 14 | Shortening | ne-sw | 48 km | 2537 | 45-25 Ma | 13, 24 |
| | 16 | Shortening | ne-sw | 39 km | 2534 | 20-10 Ma | 16, 17 |
| | 18 | Shortening | ene-wsw | 43 km | 2529.2531 | 45-15 Ma | 18 |
| | 19 | Shortening | e-w | 96 km | 2524 | 25-15 Ma | 16, 19 |
| | 21 | Shortening | e-w | 62 km | 2520 | 20-10 Ma | 25, 26, 27 |
| **Altiplano** | 9 | Shortening | ne-sw | 15 km | 2597 | 15-0 Ma | 9 |
| | 14 | Shortening | ne-sw | 40 km | 2542 | 15-0 Ma | 13 |
| | 16 | Shortening | ne-sw | 47 km | 2587.2598 | 15-0 Ma | 16 |
| | 19 | Shortening | ne-sw | 41 km | 2526 | 15-0 Ma | 16 |
| | 20 | Shortening | e-w | 24 km | 2526 | 33-27 Ma | 31 |
| | 20 | Shortening | e-w | 41 km | 2526 | 15-0 Ma | 31 |
| **Precordillera** | 29 | Shortening | e-w | 95 km | 2505 | 13-0 Ma | 32, 33 |
| | 31 | Shortening | e-w | 88 km | 2505 | 13-0 Ma | 33, 34 |
| **Principal Cordillera** | 29 | Shortening | e-w | <30 km | 2513 | 20-8,6 Ma | 32, 35 |
| | 32 | Shortening | e-w | 55-60 km | 2511 | 20-8,6 Ma | 35, 36 |
| | 33 | Shortening | e-w | 55-60 km | 2512 | 20-8,6 Ma | 35, 36 |
| | 38 | Shortening | e-w | 73 km | 2507 | 20-8,6 Ma | 35, 36 |

| | | | | | | | |
|---|---|---|---|---|---|---|---|
| | 39 | Shortening | e-w | 29,5 km | 2591 | 11,6-0 Ma | 37 |
| | 39 | Extension | e-w | 3 km | 2591 | 33,9-16 Ma | 37 |
| | 40.41 | Shortening | e-w | 5,9-12,5 km | 2589 | 100,5-56 Ma | 37 |
| | 40.41 | Extension | e-w | 0,8-1 km | 2589 | 56-11,6 Ma | 37 |
| | 40.41 | Shortening | e-w | 5,3-13,2 km | 2589 | 11,6-0 Ma | 37 |
| | 42 | Shortening | e-w | 24 km | 2502 | 11,6-0 Ma | 37, 38 |
| **Frontal Cordillera** | 30 | Shortening | e-w | 8 km | 2518 | 8,5-4 Ma | 39, 40 |
| | 32.33 | Shortening | e-w | 18 km | 2510 | 8,5-4 Ma | 39, 40 |
| | 34.35 | Shortening | e-w | 15-23 km | 2509 | 8,5-4 Ma | 40, 41 |
| | 36.37 | Shortening | e-w | 8 km | 2506 | 7,7-4 Ma | 41 |
| **Santa Barbara System** | 23 | Shortening | e-w | 30 km | 2517 | 26,7 Ma | 27, 42 |
| | 24 | Shortening | e-w | 21 km | 2517 | 26,7 Ma | 27, 28 |
| | 25 | Shortening | e-w | 21 km | 2517 | 26,7 Ma | 27, 42 |
| **Puna** | 26 | Shortening | e-w | 37 km | 2522 | 37,8-0 Ma | 43 |
| **Patagonian Cordillera** | 43 | Shortening | e-w | 35 km | 2586 | 11,6-5,3 Ma | 44 |
| | 44 | Shortening | e-w | 29 km | 2585 | 11,6-5,3 Ma | 45 |
| | 45 | Shortening | ne-sw | 27 km | 2584 | 88-74 Ma | 46 |
| | 45 | Shortening | ne-sw | 6.1 km | 2584 | 74-27 Ma | 46 |
| | 45 | Shortening | ne-sw | 3.4 km | 2584 | 27-21 Ma | 46 |
| | 45 | Shortening | ne-sw | 2.2 km | 2584 | 21-18 Ma | 46 |
| | 45 | Shortening | ne-sw | 0.9 km | 2584 | 18-0 Ma | 46 |
| | 46 | Shortening | ne-sw | ~23 km | 2583 | 84-33,9 Ma | 47 |
| | 47 | Shortening | ne-sw | ~23 km | 2584 | 84-33,9 Ma | 47 |
| | 48 | Shortening | ne-sw | ~23 km | 2585 | 84-33,9 Ma | 47 |
| | 49 | Shortening | ne-sw | 50-83 km | 2580 | 66-0 Ma | 48 |
| | 50 | Shortening | n-s | 50-83 km | 2581 | 66-0 Ma | 38 |
| **Sierras Pampeanas** | 28 | Shortening | e-w | 20 km | 2504 | 10-0 Ma | 49, 50, 51 |

| | | | | | | | |
|---|---|---|---|---|---|---|---|
| | 29 | Shortening | e-w | 10-20 km | 2504 | 10-0 Ma | 36, 51 |
| **Magallanes-Fagnano Fault system** | | Strike slip | left lateral | 50 km | | 8-0 Ma | 52 |
| **Western Cordillera and coastal belt** | 5 | Shortening | ne-sw | 52,5 km | 2555 | 50-0 Ma | 6 |
| | 7 | Shortening | ne-sw | 80 km | 2548 | 50-0 Ma | 6 |
| **Subduction erosion** | Between 22-27 | Subduction erosion | | ~31 km | 2527, 2516 | 20-0 Ma | 53, 54 |
| | Between 22-28 | Subduction erosion | | ~31 km | 2527, 2516 | 20-0 Ma | 55 |
| | 29 | Subduction erosion | | ~40 km | 2514 | 15-0 Ma | 56 |
| | 33 | Subduction erosion | | ~40 km | 2512 | 10-0 Ma | 53 |
| | 41 | Subduction erosion | | ~10 km | 2503 | 11-3 Ma | 53 |

## Supplementary references


1. Gil Rodriguez, W., Baby, P. & Ballard, J.-F. Structure et contrôle paléogéographique de la zone subandine péruvienne. *Comptes Rendus de l'Academie des Sciences de Paris* **333,** 741–748 (2001).
2. Aleman, A. M. & Marksteiner, R. Structural styles in the Santiago fold and thrust belt, Peru: A salt related orogenic belt. *Second ISAG* 147–153 (1993). doi:10.1130/0091-7613(1998)026<0723:TSACTI>2.3.CO;2
3. Kley, J. & Monaldi, C. R. Tectonic shortening and crustal thickness in the Central Andes: How good is the correlation? *Geology* **26,** 723–726 (1998).
4. Hermoza, W. *et al.* The Huallaga foreland basin evolution: Thrust propagation in a deltaic environment, northern Peruvian Andes. *J. South Am. Earth Sci.* **19,** 21–34 (2005).
5. Eude, A. La croissance des Andes centrales du nord du Pérou (5-9°S): Propagation d'un prisme orogénique dans un contexte d'héritage tectonique et de subduction plane. (Université Toulouse III Paul Sabatier, 2014).
6. Pfiffner, O. & Gonzalez, L. Mesozoic–Cenozoic Evolution of the Western Margin of South America: Case Study of the Peruvian Andes. *Geosciences* **3,** 262–310 (2013).
7. Koch, E. *Die Tektonik im Subandin des Mittel-Ucayali-Gebietes, Ostperu*. (1962). doi:10.3390/geosciences3020262
8. Espurt, N. *et al.* Paleozoic structural controls on shortening transfer in the Subandean foreland thrust system, Ene and southern Ucayali basins, Peru. *Tectonics* **27,** n/a–n/a (2008).

9. Gotberg, N., McQuarrie, N. & Caillaux, V. C. Tectonics and uplift in Central Andes (Peru, Bolivia and northern Chile) from Eocene to present. *Geological Society of America Bulletin* **122,** 727–742 (2009).
10. Sébrier, M., Lavenu, A., Fornari, M. & Soulas, J. P. Tectonics and uplift in Central Andes (Peru, Bolivia and northern Chile) from Eocene to present. *Géodynamique* (1988).
11. Roeder, D. Andean-age structure of Eastern Cordillera (Province of La Paz, Bolivia). *Tectonics* **7,** 23–39 (1988).
12. Roeder, D. & Chamberlain, R. L. Structural geology of sub-Andean fold and thrust belt in northwestern Bolivia. *AAPG Special Volumes* **62,** 459–479 (1995).
13. McQuarrie, N., Barnes, J. B. & Ehlers, T. A. Geometric, kinematic and erosional history of central Andean Plateau (15-17°S), northern Bolivia. *Tectonics* **27,** TC3007–doi:10.1029–2006TC002054 (2008).
14. Baby, P., Hérail, G., Lopez, J. M. & López, O. Structure de la zone subandine de Bolivie: influence de la géométrie des séries sédimentaires antéorogéniques sur la propagation des chevauchements. *Comptes Rendus Acadademie Science Paris* (1989).
15. Baby, P., Rochat, P., Mascle, G. & Hérail, G. Neogene shortening contribution to crustal thickening in the back arc of the Central Andes. *Geology* **25,** 883–886 (1997).
16. McQuarrie, N. The kinematic history of the central Andean fold-thrust belt, Bolivia: implications for building a high plateau. *Geological Society of America Bulletin* **114,** 950–963 (2002).
17. Eichelberger, N. *et al.* New constraints on the chronology, magnitude, and distribution of deformation within the central Andean orocline. *Tectonics* **32,** 1432–1453 (2013).
18. Barnes, J. B., Ehlers, T. A., McQuarrie, N., O'Sullivan, P. B. & Tawackoli, S. Thermochronometer record of central Andean Plateau growth, Bolivia (19.5°S). *Tectonics* **27,** TC3003–doi:10.1029–2007TC002174 (2008).
19. Dunn, J. F., Hartshorn, K. G. & Hartshorn, P. W. Structural styles and hydrocarbon potential of the sub-Andean thrust belt of southern Bolivia. *AAPG Special Volumes* **62,** 523–543 (1995).
20. Uba, C. E., Kley, J., Strecker, M. R. & Schmitt, A. K. Unsteady evolution of the Bolivian Subandean thrust belt: The role of enhanced erosion and clastic wedge progradation. *Earth and Planetary Science Letters* **281,** 134–146 (2009).
21. Echavarria, L., Hernández, R., Allmendinger, R. & Reynolds, J. Subandean thrust and fold belt of northwestern Argentina: Geometry and timing of the Andean evolution. *AAPG Bulletin* **87,** 965–985 (2003).
22. Arriagada, C., Roperch, P., Mpodozis, C. & Cobbold, P. R. Paleogene building of the Bolivian Orocline: Tectonic restoration of the central Andes in 2-D map view. *Tectonics* **27,** n/a–n/a (2008).
23. Barnes, J. B., Ehlers, T. A., McQuarrie, N., O'Sullivan, P. B. & Pelletier, J. D. Eocene to recent variations in erosion across the central Andean fold-thrust belt, northern Bolivia: Implications for plateau evolution. *Earth and Planetary Science Letters* **248,** 118–133 (2006).
24. Barnes, J. B., Ehlers, T. A., Insel, N., McQuarrie, N. & Poulsen, C. J. Linking orography, climate, and exhumation across the central Andes. *Geology* **40,** 1135–1138 (2012).
25. Kley, J. Transition from basement-involved to thin-skinned thrusting in the Cordillera Oriental of southern Bolivia. *Tectonics* **15,** 763–775 (1996).
26. Müller, J. P., Kley, J. & Jacobshagen, V. Structure and Cenozoic kinematics of the Eastern Cordillera, southern Bolivia (21°S). *Tectonics* **21,** 1–1–1–24 (2002).

27. Ege, H., Sobel, E. R., Scheuber, E. & Jacobshagen, V. Exhumation history of the southern Altiplano plateau (southern Bolivia) constrained by apatite fission track thermochronology. *Tectonics* **26,** n/a–n/a (2007).
28. Pearson, D. M. *et al.* Influence of pre-Andean crustal structure on Cenozoic thrust belt kinematics and shortening magnitude: Northwestern Argentina. *Geosphere* **9,** 1766–1782 (2013).
29. Grier, M. E., Salfity, J. A. & Allmendinger, R. W. Andean reactivation of the Cretaceous Salta rift, northwestern Argentina. *Journal of South American Earth …* **4,** 351–372 (1991).
30. Cristallini, E., Cominguez, A. H. & Ramos, V. A. Deep structure of the Metan-Guachipas region: tectonic inversion in Northwestern Argentina. *J. South Am. Earth Sci.* **10,** 403–421 (1997).
31. Elger, K., Oncken, O. & Glodny, J. Plateau-style accumulation of deformation: Southern Altiplano. *Tectonics* **24,** n/a–n/a (2005).
32. Allmendinger, R. W. *et al.* Foreland shortening and crustal balancing in the Andes at 30°S latitude. *Tectonics* **9,** 789–809 (1990).
33. Allmendinger, R. W. & Judge, P. A. The Argentine Precordillera: A foreland thrust belt proximal to the subducted plate. *Geosphere* **10,** 1203–1218 (2014).
34. Gosen, Von, W. Structural evolution of the Argentine precordillera: the Rio San Juan section. *Journal of Structural Geology* **14,** 643–667 (1992).
35. Ramos, V. A., Cristallini, E. O. & Pérez, D. J. The Pampean flat-slab of the Central Andes. *J. South Am. Earth Sci.* **15,** 59–78 (2002).
36. Ramos, V. A., Cegarra, M. & Cristallini, E. Cenozoic tectonics of the High Andes of west-central Argentina (30–36°S latitude). *Tectonophysics* **259,** 185–200 (1996).
37. Rojas Vera, E. A., Folguera, A., Zamora Valcarce, G., Bottesi, G. & Ramos, V. A. Structure and development of the Andean system between 36° and 39°S. *Journal of Geodynamics* **73,** 34–52 (2014).
38. Kley, J., Monaldi, C. R. & Salfity, J. A. Along-strike segmentation of the Andean foreland: causes and consequences. *Tectonophysics* **301,** 75–94 (1999).
39. Heredia, N., Rodríguez Fernández, L. R., Gallastegui, G., Busquets, P. & Colombo, F. Geological setting of the Argentine Frontal Cordillera in the flat-slab segment (30°00′–31°30′S latitude). *J. South Am. Earth Sci.* **15,** 79–99 (2002).
40. Giambiagi, L. B. & Ramos, V. A. Structural evolution of the Andes in a transitional zone between flat and normal subduction (33 30′–33 45′ S), Argentina and Chile. *J. South Am. Earth Sci.* **15,** 101–116 (2002).
41. Giambiagi, L., Mescua, J., Bechis, F., Tassara, A. & Hoke, G. Thrust belts of the southern Central Andes: Along-strike variations in shortening, topography, crustal geometry, and denudation. *Geological Society of America Bulletin* **124,** 1339–1351 (2012).
42. Kley, J. & Monaldi, C. R. Tectonic inversion in the Santa Barbara System of the central Andean foreland thrust belt, northwestern Argentina. *Tectonics* **21,** 11–1–11–18 (2002).
43. Coutand, I. *et al.* Style and history of Andean deformation, Puna plateau, northwestern Argentina. *Tectonics* **20,** 210–234 (2001).
44. Kraemer, P. E. Structure of the Patagonian Andes: Regional Balanced Cross Section at 50° S, Argentina. *International Geology Review* **40,** 896–915 (1998).
45. Ghiglione, M. C. *et al.* Geodynamic context for the deposition of coarse-grained deep-water axial channel systems in the Patagonian Andes. *Basin Research* **26,** 726–745 (2014).
46. Fosdick, J. C. *et al.* Kinematic evolution of the Patagonian retroarc fold-and-thrust belt and Magallanes foreland basin, Chile and Argentina,

51 30'S. *Geological Society of America Bulletin* **123,** 1679–1698 (2011).
47. Betka, P. M. Structure of the Patagonian fold-thrust belt in the Magallanes region of Chile, 53° - 55° S Lat. (University of Texas at Austin, 2013).
48. Klepeis, K. *et al.* Continental underthrusting and obduction during the Cretaceous closure of the Rocas Verdes rift basin, Cordillera Darwin, Patagonian Andes. *Tectonics* **29,** n/a–n/a (2010).
49. Allmendinger, R. W. Tectonic development, southeastern border of the Puna Plateau, northwestern Argentine Andes. *Geological Society of America Bulletin* **97,** 1070 (1986).
50. Jordan, T. E. & Allmendinger, R. W. The Sierras Pampeanas of Argentina; a modern analogue of Rocky Mountain foreland deformation. *American Journal of Science* **286,** 737–764 (1986).
51. Coughlin, T. J., O'Sullivan, P. B., Kohn, B. P. & Holcombe, R. J. Apatite fission-track thermochronology of the Sierras Pampeanas, central western Argentina: Implications for the mechanism of plateau uplift in the Andes. *Geology* **26,** 999 (1998).
52. Torres-Carbonell, P. J., Olivero, E. B. & Dimieri, L. V. Control en la magnitud de desplazamiento de rumbo del Sistema Transformante Fagnano, Tierra del Fuego, Argentina. *Rev. geol. Chile* **35,** 63–77 (2008).
53. Kukowski, N. & Oncken, O. in *The Andes* 217–236 (Springer Berlin Heidelberg, 2006). doi:10.1007/978-3-540-48684-8_10
54. Huene, von, R. & Ranero, C. R. Subduction erosion and basal friction along the sediment-starved convergent margin off Antofagasta, Chile. *J Geophys Res* **108,** (2003).
55. Scheuber, E., Bogdanic, T., Jensen, A. & Reutter, K.-J. in *Tectonics of the Southern Central Andes* 121–139 (Springer Berlin Heidelberg, 1994). doi:10.1007/978-3-642-77353-2_9
56. Stern, C. R. Role of subduction erosion in the generation of Andean magmas. *Geology* **19,** 78 (1991).
